# Low post-arthroplasty infection rate is possible in developing countries: long-term experience of local vancomycin use in Iran

**DOI:** 10.1186/s13018-021-02344-2

**Published:** 2021-03-17

**Authors:** Mohammad Naghi Tahmasebi, Arash Sharafat Vaziri, Fardis Vosoughi, Mohamad Tahami, Majid Khalilizad, Hamid Rabie

**Affiliations:** 1 Knee, Sport and Reconstruction Surgery, Knee Surgery Fellowship Program, Orthopaedic Surgery Department, Shariati Hospital and School of Medicine, Tehran University of Medical Sciences, Tehran, Iran; 2 Fellowship of Knee, Sport and Reconstruction Surgery, Department of Orthopaedic and Trauma Surgery, Shariati Hospital, Tehran University of Medical Sciences, Tehran, Iran; 3 Department of Orthopaedic and Trauma Surgery, Shariati Hospital and School of Medicine, Tehran University of Medical Sciences, Tehran, Iran; 4 Fellowship of Knee, Sport and Reconstruction Surgery, Department of Orthopaedic Surgery, Chamran Hospital, Shiraz University of Medical Sciences, Shiraz, Iran; 5 Bone and Joint Research Center, Shiraz University of Medical Sciences, Shiraz, Iran; 6 Department of Orthopaedic Surgery, Shahid Beheshti Hospital, Babol University of Medical Sciences, Babol, Mazandaran Iran

**Keywords:** Vancomycin, Total knee arthroplasty, Prosthetic infection, Superficial wound infection, Antibiotic therapy

## Abstract

**Background:**

Utilizing intrawound vancomycin powder in TKA surgery has yielded rather contrasting results in the current literature. Furthermore, CDC criteria, although effective in general, are not specifically designed for post-TKA infections. Here, we present a 7-year experience of vancomycin use in primary TKA in a high-volume tertiary knee center in Iran. Also, new criteria are proposed to detect suspected superficial post-TKA infections.

**Methods:**

This is a retrospective analysis of primary total knee arthroplasties performed in a tertiary knee center, from March 2007 to December 2018, by a single senior knee surgeon. All patients with follow-up periods of less than 1 year were excluded from the study. Since March 2011, all patients received vancomycin (powder, 1 g) before water-tight closure of the joint capsule. A comparison was made between this group and historical control subjects (operated from March 2007 to March 2011).

**Results:**

Altogether, 2024 patients were included in the study. The vancomycin and the control groups included 1710 and 314 cases respectively. Patients were mostly women (male to female ratio, 1 to 4), with a mean age of 65.20 (SD = 10.83) years. In the vancomycin group, the rate of suspected SII (1.87%) and PJI (0.41%) was significantly lower than the control group (*P* = 0.002).

**Conclusions:**

Our experience shows that application of local vancomycin during TKA surgery could be a reasonable infection prevention measure, although prospective randomized studies are required to evaluate its efficacy.

## Background

Joint replacement enhances function and alleviates pain in a completely destructed knee, but requires implementing high technical perfection by surgeons or it could result in devastating consequences [1]. Deep periprosthetic joint infection (PJI) following total knee arthroplasty (TKA) usually needs treatment by two-stage revision arthroplasty [2, 3]. It causes significant bone loss, as well as substantial injury to soft tissue structures, and could necessitate higher-constraint prostheses with lower reported survivals [4]; therefore, even the lowest infection rate is too high to be accepted by the surgeon or the patient after TKA and reducing infection rate has always been a challenge. This is especially true in Iran, where heavy economic sanctions have caused revision devices and prostheses less available [3–7]. Thus, PJI after TKA is an intolerable complication in Iran.

Considerable research has been carried out for the prevention and also early detection of post-TKA infections [2, 5, 6, 8–11]. Regarding prevention, a recent topic is utilizing intrawound vancomycin powder. Separate in vivo rat investigations [12, 13] have demonstrated the effectiveness of intrawound antibiotics in clearing *Staphylococcus aureus* from contaminated femoral implants. Intraoperative use of vancomycin powder has also been shown to be effective in reducing the chance of infection in spine surgeries [14–16], but literature on its use in TKA surgery has yielded rather contrasting results [17–19].

Regarding early detection, the latest consensus on PJI has provided some valuable criteria to diagnose a deep infection [20], but literature on the detection of superficial infection after TKA is not clear [21, 22]. Some studies [22] have used the US Centers for Disease Control and Prevention (CDC) criteria for identifying surgical site infections which were defined by the CDC in 1992 as those occurring within 30 days of surgery [23]. CDC criteria although effective in general are not specifically designed for post-TKA infections. In fact, most TKA patients experience pain, tenderness, and swelling at the surgical site. Furthermore, it does not seem logical to wait for wound drainage and positive cultures to intervene.

In the current study, superficial incisional infections (SIIs) were suspected, in case of any signs of erythema and/or warmth and/or itching and/or increased local pain on the surgical site. We present a 7-year experience of vancomycin use in a high-volume tertiary knee center in Iran and we set out to report our findings as well as discuss the reasonable explanations.

## Methods

This is a retrospective analysis of primary total knee arthroplasties performed in a tertiary knee center, from March 2007 to December 2018. The study was approved by the Institutional Review Board of Tehran University of Medical Sciences. Informed written consent was obtained from the patients. Intrawound vancomycin was used since March 2011 in all TKAs. The arthroplasties performed prior to 2011 were considered as the historical control group. Other infection prevention protocols and surgical technique were the same between the two groups. All arthroplasties were performed by a single senior knee surgeon. Cases with follow-up periods of less than 1 year were excluded. Records of each patient’s age, gender, operation time, and length of stay in the hospital after surgery were collected. Rates of superficial and deep infection are reported along with a detailed report on comorbidities of the infected cases.

**Fig. 1:**
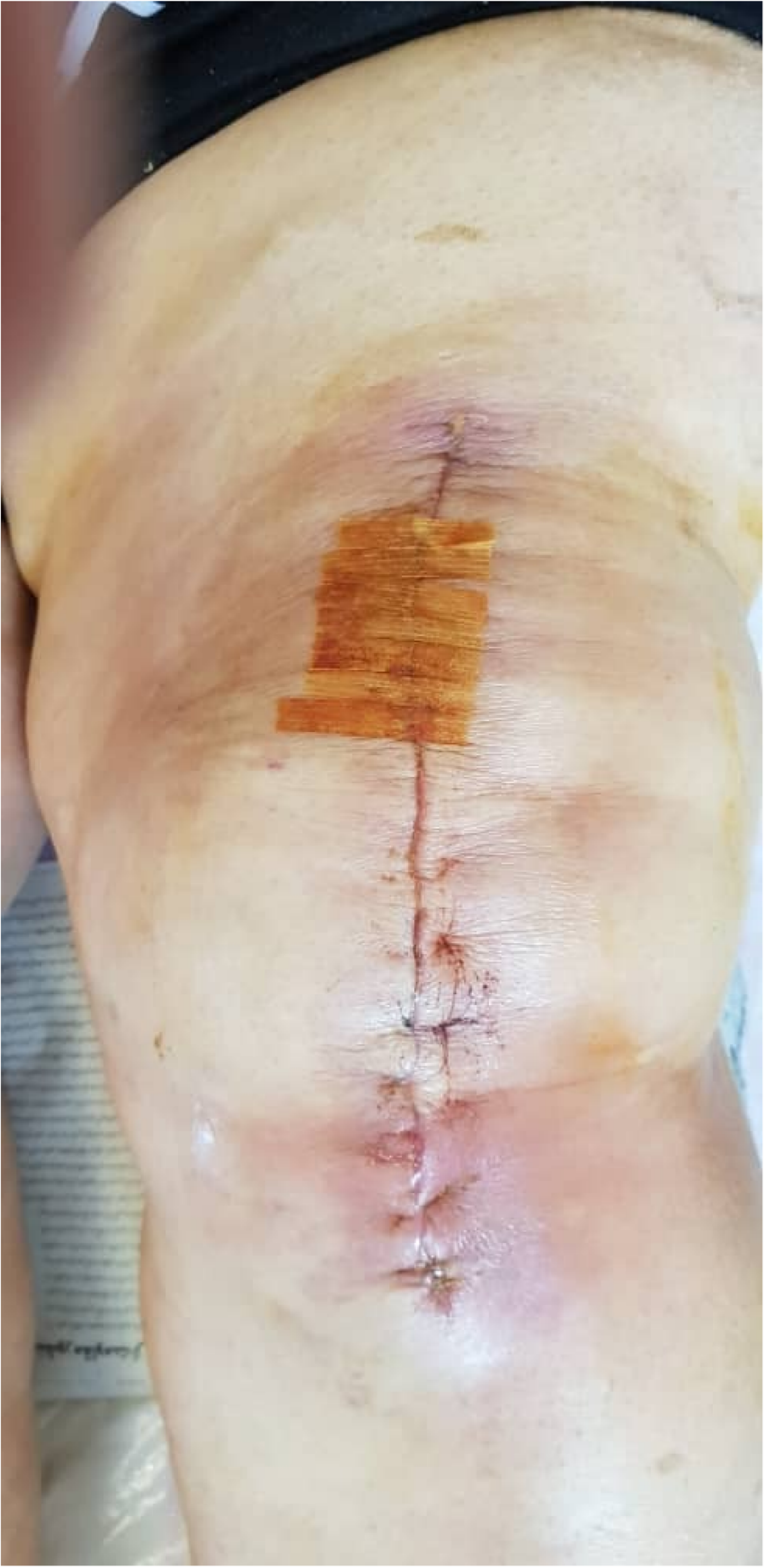
Photography of the surgical wound, 10 days after a left side TKA. The patient exhibited local wound erythema and warmth with no discharge and no signs of joint involvement. All symptoms subsided after a 1-week trial of oral antibiotic therapy [this clinical photograph was taken by Dr. M.N.T. for the sole purpose of the current study. Written informed consent was obtained from the patient]

We classified all patients with any of the following 4 criteria including erythema and/or warmth and/or itching and/or increased local pain at the surgical wound as suspected cases of SII (Fig. 1) and treated them with a 1-week course of oral levofloxacin 500 mg twice daily. None of them was proved to be infections, as no culture sample was analyzed. In this retrospective analysis, all PJI cases met the criteria proposed by the proceedings of the Philadelphia consensus [20].

**Fig. 2:**
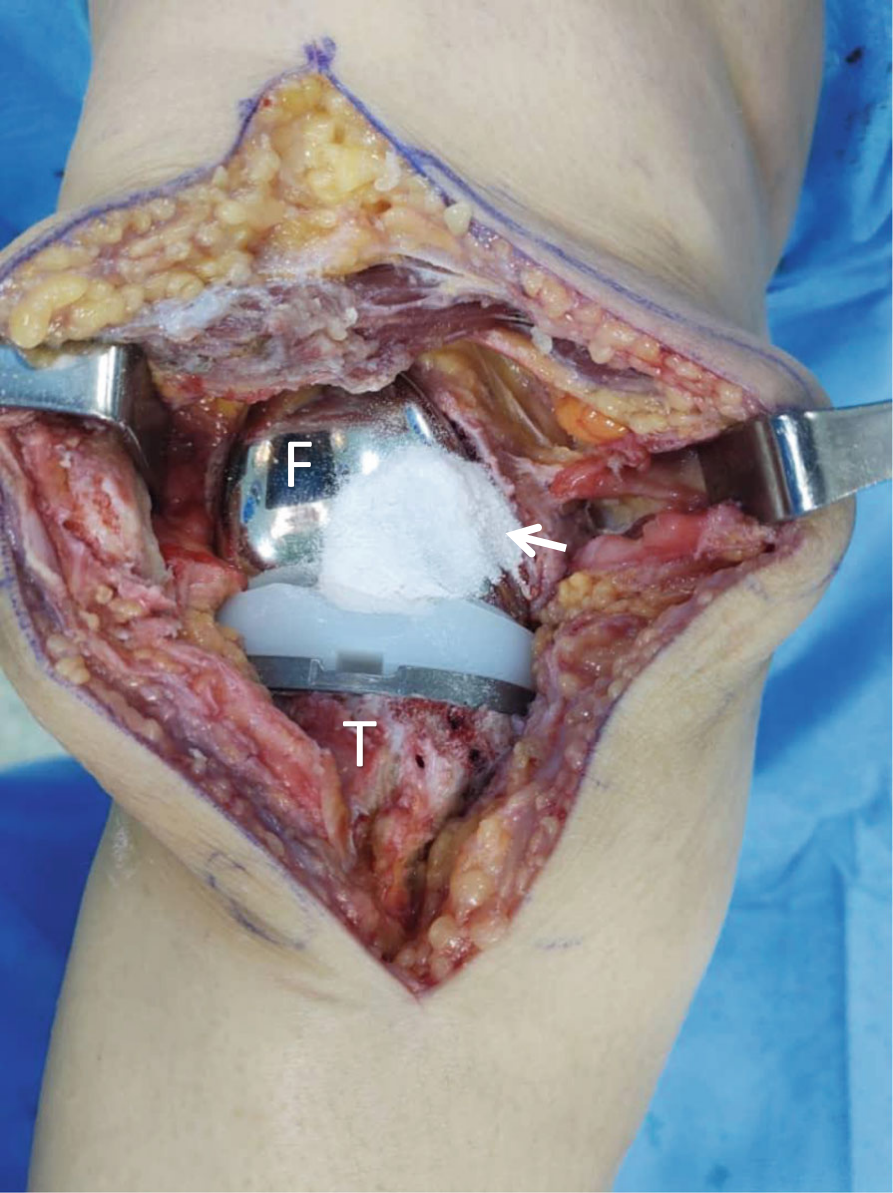
Intraoperative photography of a right side TKA surgical wound after application of local vancomycin right before tightly closing the joint capsule. *F* femoral component, *T* tibia. Arrow: vancomycin powder [this intraoperative photograph was taken by Dr. F.V. for the sole purpose of the current study. Written informed consent was obtained from the patient]

All patients underwent cemented TKAs without patellar replacement (NexGen, Zimmer Biomet prostheses until July 2017 and Persona Zimmer Biomet prostheses since then). As an infection prevention measure since March 2011, intrawound vancomycin (powder, 1 g) was used at the end of TKA surgeries right before tightly closing the joint capsule (Fig. 2). It is worth mentioning that in addition to intrawound vancomycin, intra-articular injection of tranexamic acid was also performed as a routine practice in all cases since 2007, and its use was previously published from this center [24]. Data are reported as means, standard deviations (SD), and percentages. Comparing percentages was performed by the StatPac software (StatPac Inc. 2017). A two-sample *t* test was used to compare means. Statistical analysis was performed using SPSS software version 25 (IBM corporation). Significance was defined as a *P* value under 0.05.

## Results

Amidst a total of 2494 TKAs performed during the study time period, 2024 patients had a minimal documented follow-up period of 1 year and were included in the study. Demographic data are depicted in Table 1. Patients were mostly women (male to female ratio, 1 to 4), with a mean age of 65.20 (SD = 10.83) years. Demographic characteristics were the same in the control (*n* = 314) and the vancomycin groups (*n* = 1710).

**Table 1:** General preoperative characteristics of the patients

Characteristics	Number of cases	Number of controls	*P* value
Female (%)	1393 (81.45)	252 (80.25)	0.50
Mean age (SD)	64.99 (11.49)	66.37 (8.90)	0.18
Mean length of hospital stay (SD)	3.13 (4.07)	3.76 (4.91)	0.68
Mean operation time (SD)	60.30 (44.19)	62.23 (39.51)	0.12
Superficial incisional infection (%)	32 (1.87)	36 (11.46)	0.00
Deep joint infection (%)	7 (0.41)	6 (1.91)	0.00

### Control group

Among the 36 patients (11.46%) who showed signs of suspected superficial incisional infection and were treated with 1 week of oral levofloxacin, none lead to deep infection. Meanwhile, deep infection occurred in 6 patients (1.91%). One of them was early PJI (3 weeks after surgery) and underwent irrigation and polyethylene exchange. The other 5 were late PJI and underwent 2-stage revision surgery.

### Vancomycin group

Taking both suspected and definite infections into account, the overall infection rate was 2.16% (37 of 1710 patients), including 7 periprosthetic deep joint infections and 32 suspected SIIs. Besides, from the 32 suspected superficial infections that were treated with oral antibiotics, all reported disappearance of symptoms yet 2 cases returned with late PJI. Except for 7 cases, all cases of suspected SII were detected close to 1 month after surgery. A detailed report on the comorbidities of the patients who developed superficial infection is provided in Table 2. The most common characteristics among this group of patients were age > 60 years, followed by obesity and diabetes mellitus in order of prevalence. Except for one, every one of them had at least one comorbidity. All PJI cases were females. There were no cases of early PJIs defined as those occurring over the first 6 weeks after surgery. Thereupon, no polyethylene exchange was performed over the studied time frame. All in all, 7 patients (0.41%) underwent two-stage knee arthroplasty due to deep joint infections.

**Table 2:** Comorbidities of the 32 patients with SII in the vancomycin group. Cases who finally developed deep infection are marked with an asterisk. *DM* diabetes mellitus, *RA* rheumatoid arthritis, *KD* kidney disease, *Obesity* a body mass index more than 30 kg/m^2^

Case no.	DM	RA	KD	Obesity	Age > 60 years
1	+	−	+	+	+
2	+	−	+	+	+
3	+	−	−	+	+
4	+	−	−	+	+
5	+	−	−	+	+
6*	−	+	−	+	+
7	−	−	−	+	+
8	+	−	−	−	−
9	−	−	−	+	+
10	+	−	−	+	+
11	+	−	−	+	+
12	+	−	−	+	+
13	−	+	−	−	+
14	+	−	−	+	+
15	+	−	−	+	+
16	−	−	−	+	+
17	+	−	−	−	+
18	+	−	−	+	+
19	+	−	−	+	+
20	+	−	−	+	+
21	+	−	−	+	+
22	−	+	−	+	+
23	+	−	−	+	+
24*	+	−	−	+	+
25	+	−	−	+	+
26	−	−	−	−	+
27	−	−	−	+	+
28	−	−	−	−	+
29	+	−	−	+	+
30	+	−	−	+	+
31	+	−	−	+	+
32	+	−	−	+	+
Total	23	3	2	27	31

### Comparison

Regarding baseline characteristics, there was no significant difference among the groups (Table 1). In the historical control subjects, the rate of suspected SII (11.46%) and PJI (1.91%) was significantly higher than the vancomycin group (*P* = 0.002).

## Discussion

This is a retrospective analysis of an 11-year experience of a single knee surgeon from a referral knee center, and a total of 2024 consecutive primary TKAs were enrolled. Patients include those from all over Iran. Intrawound vancomycin powder was used in 1710 cases, and the results were compared with a historical control group with 314 cases. The mean age, mean duration of surgery, and mean length of stay along with data on comorbidities and final outcome of those who were diagnosed to have superficial or deep infections are presented.

The mean length of stay in our study was 3.21 days (SD = 2.05), which is somewhat higher than recent reports [25, 26]. In our center, preoperative planning and paraclinical work-ups of patients were performed on an outpatient basis. Only after determining precise surgical plans, having prepared required devices, and optimizing their medical states were TKA candidates scheduled for surgery. Patients were admitted to the hospital early in the morning and then underwent surgery the same day, and if alert and well, patients were discharged the next morning. The only exceptions were those patients coming from distant cities or rural places of the country who neither had a place to stay in Tehran nor could come back any time sooner than 2 months after surgery. Since a strong family physician-based postop care system is not available in Iran, this small group of patients was discharged after 1 to 2 weeks, until knee range of motion was secured and the surgical wound completely healed. Of our TKAs, 90% were discharged in less than 6 days and 60% were discharged in less than 3 days after surgery.

The mean age in our study population was 65.20 (SD = 10.83), which is somewhat younger than that of similar studies [22], despite the fact that people here refer for surgery at the latest stages of knee joint destruction. This obvious lower age of TKA candidates in Iran could be due to the Middle Eastern lifestyle which is characterized by high-flexion activities such as praying, sitting on the ground, and using Iranian toilets which need squatting.

Previously, it has been proved that increased operative time could result in a higher complication rate especially infection, venous thromboembolism (VTE), and patient dissatisfaction [25, 27–32]. Each TKA surgery took an average of 60.30 min (SD = 44.19) from incision to wound dressing, which is comparable to other reports [28, 33].

In the vancomycin group, there were 32 cases (1.87%) of suspected superficial incisional infection and no cases of early PJI. Seven cases of late PJI (0.41%) were diagnosed and underwent two-stage revision arthroplasties, among whom 2 patients had a history of being treated for suspected SII. Our rate of PJI is in concert with recent reported rates [22].

A discussion needs to be made on how to diagnose superficial incisional infections after TKA. To the best of our knowledge, there is no unified approach in the literature on this matter and CDC criteria are not designed, specifically for post-TKA infections. Our approach was to detect all cases suspected of having a superficial incisional infection and treating them with a short course of antibiotics. We cannot be sure if these 32 cases were in fact infections or solely inflammations or hypersensitivity reactions to Monocryl stitches. There was a 6-month period of time from April 2016 to September 2016, when an increase in these cases was observed (10 cases). After intense scrutiny, we found similar reports from other centers in Tehran using the same brand of Monocryl as ours. After switching to rapid Vicryl, the SII rate declined. The role of stitches in TKA wound complications has been reported before [34]. We cannot still be sure how many suspected SIIs were actually stitch reactions. Howbeit, the fact that most cases happened close to 1 month after surgery (the time that Vicryl starts to resorb) makes this explanation more convincing. Indeed, it seems that even the quality of stitches we used has been seriously affected by tight economic sanctions. There were no records of SII over the last year of the study time.

There were some key routine practices that possibly prevented higher infection rates in our study.

First and foremost, it is the oriented and highly trained operating room (OR) personnel who understood the importance of infection control and strict infection prevention standards. Second is our approach to early wound infection. During postop follow-up examinations, if the surgical wound showed signs of erythema and/or warmth and/or itching and/or increased local pain at the surgical wound, a 1-week course of oral levofloxacin 500 mg twice daily was prescribed. We do not use CDC criteria for surgical site infection, as it is not specifically designed for knee arthroplasty. Pain, tenderness, and/or swelling which are mentioned in CDC criteria occur in the early postoperative period of most TKAs and do not seem specific enough to diagnose a superficial infection. In fact, in knee arthroplasty, due to cumbersome management and poor outcomes of PJI, we need early diagnosis of any superficial infection and wound drainage or positive culture mentioned in the CDC criteria would procrastinate diagnosis and prevent early intervention. The third probable factor could be the use of intrawound vancomycin powder before water-tight closure of the joint capsule. Since we started routine use of vancomycin powder in the wound at the end of TKA surgeries, the infection rate decreased significantly (*P* = 0.002). The effect of vancomycin powder on infection rate has not been proved in TKA (17-19), in contrast to spine surgery where it has been well established [14–16]. Although we report promising results, its effectiveness cannot be proved based on the current study either.

The strengths of this study include a large sample size and enrolling consecutive patients to decrease selection bias. Also, all the arthroplasties were performed by a single surgeon with a unified surgical technique, which eliminates the confounding effect of different surgical techniques (Table 3). Nevertheless, there is an inherent probability of selection bias due to implementing historical control subjects and lack of randomization [35]. Despite our results, determining the exact effect of intrawound vancomycin on the rate of deep infection after TKA needs prospective randomized controlled trials designed specifically for its use in knee arthroplasty surgery [36–38].

**Table 3:** Summary of the limitations and advantages of the current study

Limitations	Advantages
Historical controls	Large sample size
Lack of randomization	Consecutive patients
Retrospective design	Single surgeon

## Conclusion

Our experience shows that the application of local vancomycin during TKA surgery could be a reasonable infection prevention measure, although future prospective randomized clinical trials are required to evaluate its efficacy.

## Data Availability

The datasets used and/or analyzed during the current study are available from the corresponding author on reasonable request.

## Abbreviations

TKA: Total knee arthroplasty
SII: Superficial incisional site infection
PJI: Periprosthetic joint infection
CDC: US Centers for Disease Control and Prevention
OR: Operating room
VTE: Venous thromboembolism
SD: Standard deviation

## References

[1] Healy WL, Della Valle CJ, Iorio R, Berend KR, Cushner FD, Dalury DF, Lonner JH (2013). Complications of total knee arthroplasty: standardized list and definitions of the Knee Society. Clin Orthop Relat Res..

[2] Poultsides LA, Triantafyllopoulos GK, Sakellariou VI, Memtsoudis SG, Sculco TP (2018). Infection risk assessment in patients undergoing primary total knee arthroplasty. Int Orthop..

[3] Ha CW (2017). Treatment of infected total knee arthroplasty. Knee Surg Relat Res..

[4] Zimmerli W, Trampuz A, Ochsner PE (2004). Prosthetic-joint infections. N Engl J Med..

[5] Teo BJX, Yeo W, Chong HC, Tan AHC (2018). Surgical site infection after primary total knee arthroplasty is associated with a longer duration of surgery. J Orthop Surg (Hong Kong).

[6] Rhee C, Lethbridge L, Richardson G, Dunbar M (2018). Risk factors for infection, revision, death, blood transfusion and longer hospital stay 3 months and 1 year after primary total hip or knee arthroplasty. Can J Surg..

[7] Nabian MH, Vosoughi F, Najafi F, Khabiri SS, Nafisi M, Veisi J, Rastgou V, Ghamari S, Aakhashi A, Bahrami N, Naderi M, Maleki S, Yekaninejad MS (2020). Epidemiological pattern of pediatric trauma in COVID-19 outbreak: data from a tertiary trauma center in Iran. Injury..

[8] Inabathula A, Dilley JE, Ziemba-Davis M, Warth LC, Azzam KA, Ireland PH, Meneghini RM (2018). Extended oral antibiotic prophylaxis in high-risk patients substantially reduces primary total hip and knee arthroplasty 90-day infection rate. J Bone Joint Surg Am..

[9] Wyles CC, Hevesi M, Osmon DR, Park MA, Habermann EB, Lewallen DG (2019). 2019 John Charnley Award: increased risk of prosthetic joint infection following primary total knee and hip arthroplasty with the use of alternative antibiotics to cefazolin: the value of allergy testing for antibiotic prophylaxis. Bone Joint J.

[10] Gromov K, Troelsen A, Raaschou S, Sandhold H, Nielsen CS, Kehlet H, Husted H (2019). Tissue adhesive for wound closure reduces immediate postoperative wound dressing changes after primary TKA: a randomized controlled study in simultaneous bilateral TKA. Clin Orthop Relat Res..

[11] Maniar RN, Navaneedhan G, Ranvir S, Maniar AR, Dhiman A, Agrawal A (2019). What is the normal trajectory of interleukin-6 and C-reactive protein in the hours and days immediately after TKA?. Clin Orthop Relat Res..

[12] Cavanaugh DL, Berry J, Yarboro SR, Dahners LE (2009). Better prophylaxis against surgical site infection with local as well as systemic antibiotics. An in vivo study. J Bone Joint Surg Am..

[13] Edelstein AI, Weiner JA, Cook RW, Chun DS, Monroe E, Mitchell SM, Kannan A, Hsu WK, Stulberg SD, Hsu EL (2017). Intra-articular vancomycin powder eliminates methicillin-resistant S. aureus in a rat model of a contaminated intra-articular implant. J Bone Joint Surg Am..

[14] Bakhsheshian J, Dahdaleh NS, Lam SK, Savage JW, Smith ZA (2015). The use of vancomycin powder in modern spine surgery: systematic review and meta-analysis of the clinical evidence. World Neurosurg..

[15] Hey HW, Thiam DW, Koh ZS, Thambiah JS, Kumar N, Lau LL (2017). Is intraoperative local vancomycin powder the answer to surgical site infections in spine surgery?. Spine (Phila Pa 1976)..

[16] O'Neill KR, Smith JG, Abtahi AM, Archer KR, Spengler DM, McGirt MJ (2011). Reduced surgical site infections in patients undergoing posterior spinal stabilization of traumatic injuries using vancomycin powder. The Spine Journal..

[17] Hanada M, Nishikino S, Hotta K, Furuhashi H, Hoshino H, Matsuyama Y (2019). Intrawound vancomycin powder increases post-operative wound complications and does not decrease periprosthetic joint infection in primary total and unicompartmental knee arthroplasties. Knee Surg Sports Traumatol Arthrosc..

[18] Otte JE, Politi JR, Chambers B, Smith CA (2017). Intrawound vancomycin powder reduces early prosthetic joint infections in revision hip and knee arthroplasty. Surg Technol Int..

[19] Yavuz IA, Oken OF, Yildirim AO, Inci F, Ceyhan E, Gurhan U. No effect of vancomycin powder to prevent infection in primary total knee arthroplasty: a retrospective review of 976 cases. Knee Surg Sports Traumatol Arthrosc. 2019;1–6. 10.1007/s00167-019-05778-8 31728604

[20] Parvizi J, Gehrke T, Chen A (2013). Proceedings of the international consensus on periprosthetic joint infection. Bone Joint J.

[21] Wilson CJ, Georgiou KR, Oburu E, Theodoulou A, Deakin AH, Krishnan J (2018). Surgical site infection in overweight and obese total knee arthroplasty patients. J Orthop..

[22] Guirro P, Hinarejos P, Puig-Verdie L, Sánchez-Soler J, Leal-Blanquet J, Torres-Claramunt R, Monllau JC (2016). Superficial wound infection does not cause inferior clinical outcome after TKA. Knee Surg Sports Traumatol Arthrosc..

[23] Horan TC, Gaynes RP, Martone WJ, Jarvis WR, Emori TG (1992). CDC definitions of nosocomial surgical site infections, 1992: a modification of CDC definitions of surgical wound infections. Infect Control Hosp Epidemiol..

[24] Tahmasebi MN, Bashti K, Ghorbani G, Sobhan MR (2014). Intraarticular administration of tranexamic acid following total knee arthroplasty: a case-control study. Arch Bone Jt Surg..

[25] Husted H, Holm G, Jacobsen S (2008). Predictors of length of stay and patient satisfaction after hip and knee replacement surgery: fast-track experience in 712 patients. Acta Orthop..

[26] Otero JE, Gholson JJ, Pugely AJ, Gao Y, Bedard NA, Callaghan JJ (2016). Length of hospitalization after joint arthroplasty: does early discharge affect complications and readmission rates?. J Arthroplasty..

[27] Wills BW, Sheppard ED, Smith WR, Staggers JR, Li P, Shah A, Lee SR, Naranje SM (2018). Impact of operative time on early joint infection and deep vein thrombosis in primary total hip arthroplasty. Orthop Traumatol Surg Res..

[28] Naranje S, Lendway L, Mehle S, Gioe TJ (2015). Does operative time affect infection rate in primary total knee arthroplasty?. Clin Orthop Relat Res..

[29] Bohl DD, Ondeck NT, Darrith B, Hannon CP, Fillingham YA, Della Valle CJ (2018). Impact of operative time on adverse events following primary total joint arthroplasty. J Arthroplasty.

[30] Ricciardi BF, Oi KK, Daines SB, Lee YY, Joseph AD, Westrich GH (2017). Patient and perioperative variables affecting 30-day readmission for surgical complications after hip and knee arthroplasties: a matched cohort study. J Arthroplasty..

[31] Peersman G, Laskin R, Davis J, Peterson MG, Richart T (2006). Prolonged operative time correlates with increased infection rate after total knee arthroplasty. Hss j..

[32] Young SW, Mutu-Grigg J, Frampton CM, Cullen J (2014). Does speed matter? Revision rates and functional outcomes in TKA in relation to duration of surgery. J Arthroplasty.

[33] Duchman KR, Pugely AJ, Martin CT, Gao Y, Bedard NA, Callaghan JJ (2017). Operative time affects short-term complications in total joint arthroplasty. J Arthroplasty..

[34] Sah AP (2015). Is there an advantage to knotless barbed suture in TKA wound closure? A randomized trial in simultaneous bilateral TKAs. Clin Orthop Relat Res..

[35] Patel NN, Guild GN, Kumar AR (2018). Intrawound vancomycin in primary hip and knee arthroplasty: a safe and cost-effective means to decrease early periprosthetic joint infection. Arthroplasty Today..

[36] O’Toole RV, Joshi M, Carlini AR, Murray CK, Allen LE, Scharfstein DO (2017). Local antibiotic therapy to reduce infection after operative treatment of fractures at high risk of infection: a multicenter, randomized, controlled trial (VANCO study). J Orthopaedic Trauma..

[37] Heckmann ND, Mayfield CK, Culvern CN, Oakes DA, Lieberman JR, Della Valle CJ (2019). Systematic review and meta-analysis of intrawound vancomycin in total hip and total knee arthroplasty: a call for a prospective randomized trial. J Arthroplasty..

[38] Mediouni M (2019). A new generation of orthopaedic surgeons: “T-model”. Curr Orthopaedic Pract..

